# 人工智能在肺结节诊治中的应用专家共识(2022年版)

**DOI:** 10.3779/j.issn.1009-3419.2022.102.08

**Published:** 2022-04-20

**Authors:** 吴阶平医学基金会模拟医学部胸外科专委会

**Keywords:** 肺结节, 人工智能, 专家共识, Pulmonary nodule, Artificial intelligence, Expert consensus

## Abstract

低剂量计算机断层扫描用于肺癌筛查已被证实可减低肺癌特异性死亡率，肺癌筛查计划的推广带来了肺结节检出率的显著提高，对这些结节的判断也明显加重影像学科工作负荷。人工智能(artificial intelligence, AI)在协助肺结节良恶性判读及制定个体化治疗方案上体现出的有效性已得到初步验证。随着越来越多的AI产品在肺结节领域进行应用，吴阶平医学基金会模拟医学部胸外科专委会牵头组织该领域专家，参考国内外最新文献、临床研究数据，在专家共识达成统一意见并制定本共识，为国内同行更好地应用AI诊治肺结节提供参考意见。

## Abstract (en)

Low-dose computed tomography (CT) for lung cancer screening has been proven to reduce lung cancer deaths in the screening group compared with the control group. The increasing number of pulmonary nodules being detected by CT scans significantly increase the workload of the radiologists for scan interpretation. Artificial intelligence (AI) has the potential to increase the efficiency of pulmonary nodule discrimination and has been tested in preliminary studies for nodule management. As more and more artificial AI products are commercialized, the consensus statement has been organized in a collaborative effort by Thoracic Surgery Committee, Department of Simulated Medicine, Wu Jieping Medical Foundation to aid clinicians in the application of AI-assisted management for pulmonary nodules.

## 1 前言

肺癌是全世界最常见的恶性肿瘤，占恶性肿瘤发病率及死亡率的首位。肺癌筛查计划的积极推广显著提高了肺结节的检出率。人工智能(artificial intelligence, AI)在协助肺结节良恶性判读上的功能逐步获得重视。然而，目前对应用AI对肺结节进行辅助诊断及随访策略的大宗研究还比较少，为进一步提高我国肺结节规范化诊疗水平，撰写《人工智能在肺结节诊治中的应用专家共识(2022年版)》具有一定重要性。本共识是在临床实践经验基础上，结合现有文献经讨论和综述而成，仅代表吴阶平医学基金会模拟医学部胸外科专委会对一般建议的一致意见，不设证据类别，而共识等级则依据专家组内投票比例进行以下划分：一致共识，即支持意见≥80%; 基本一致共识，且争议小，即支持意见60%-80%; 无共识，且争议大，即支持意见 < 60%。随着以后更多肺结节AI相关文献发表，本共识将进一步更新。

## 2 肺结节诊断的现状与局限性

专家共识：低剂量螺旋计算机断层扫描(computed tomography, CT)筛查发现的肺结节，假阳性率较高，传统诊断方法在肺结节的诊断中，具有较强的局限性。AI在肺结节良恶性诊断方面已经取得了一定进展，但在病理分型预测、多次随访数据的综合判断、手术规划等方面，还存在很多问题亟待解决(**共识强度：一致共识**)。

低剂量螺旋CT筛查发现的肺结节，恶性率仅为10%-20%^[1]^; 在国家肺部筛查试验(National Lung Screening Trial, NLST)研究中，对于直径≥4 mm的肺结节，三轮低剂量螺旋CT筛查后假阳性率超过96.4%^[2]^。目前的指南建议使用正电子发射计算机断层显像(positron emission tomography CT, PET-CT)扫描、支气管内超声引导的经支气管活检(endobronchial ultrasound-guided transbronchial needle aspiration, EBUS-TBB)或经胸肺穿刺活检(transthoracic needle aspiration, TTNA)进行进一步评估。PET-CT在部分实性结节中的敏感性约为50%，在纯磨玻璃结节(pure ground glass nodule, pGGN)中甚至低于20%; EBUS-TBB对于20 mm或更小的结节，其敏感性仅为50%，对于5 mm-10 mm的结节，其诊断的敏感性仅为35%^[3]^。TTNA的出血风险为1%，患者依从性较低^[4]^。为了满足未满足的临床和经济需求，需要新的替代/补充、无创的结节管理方法，一方面，在结节处于肺癌早期阶段时提供及时的必要治疗; 另一方面，当结节被认为是良性时，尽量避免过度医疗。

AI在肺结节诊断中，能有效区分肺小结节和非结节，减少假阳性率，增加肺结节的检出率^[5]^。肺结节中的三维纹理特征、临床信息及CT图像数据计入支持向量机模型进行肺癌预测，可提高放射科医师诊断的敏感度与特异度。虽然AI在肺结节良恶性诊断方面已经取得了一定进展，但还存在很多问题亟待解决，包括创立数据资源共享平台，整合多中心、多参数、多模态标准化的大数据供科学研究^[6]^。

## 3 AI在肺结节识别中的作用

专家共识：AI在辅助医生进行肺结节识别方面，具有较大优势，在肺结节随访中判断良恶性具有重要价值(**共识强度：一致共识**)。AI对亚实性结节检测的假阴性率较高，仍需要人工阅片确认以减少漏诊(**共识强度：基本一致共识**)。

AI在CT检查结果中识别肺结节的灵敏度约为96.7%，高于放射科医生的78.1%，AI较高的敏感度和读片速度在肺癌筛查中具有重要价值^[7]^。AI对测量实性结节体积准确性更高，根据肺部影像报告和数据系统(lung imaging reporting and data system, Lung-RADs)和Fleischner指南，应酌情降低AI识别的灵敏度以限制假阳性检测率^[8]^。但是AI对亚实性肺结节识别的灵敏度较低，在最高灵敏度设置下最多只能检测到50%的亚实性结节^[9]^。因此在使用AI检测亚实性结节时，仍需要人工阅片确认以减少误报^[10]^。

除了用于肺结节检测，AI还可用于计算肺结节的体积并估计肺结节体积的倍增时间，基于AI的肺结节体积测量具有高度的可重复性，尤其对于最大径 < 10 mm的肺结节，与直径手动测量相比具有明显优势^[11]^。体积 < 50 mm^3^的肺结节与50 mm^3^-500 mm^3^之间且体积倍增时间(以3个月计算)超过400 d的肺结节被视为阴性筛查^[12]^。计算机辅助检测(computer aided detection, CAD)测量实性结节超过500 d的倍增时间对恶性肿瘤的诊断具有98%的阴性预测值^[13]^。但对于亚实性结节，即使可以分别估计部分实性结节的实性和非实性成分的倍增时间，体积测量软件的可靠性也较差^[13]^。

## 4 AI在肺结节良恶性鉴别诊断中的作用

专家共识：AI技术在肺结节良恶性鉴别中可为临床诊断提供辅助参考，但其准确性还无法取代人工(**共识强度：一致共识**)。融合多模态信息的肺癌诊断技术能够得到更加精确的肺癌诊断效果(**共识强度：基本一致共识**)。

根据既往综述，四项基于不同类型数据库的训练和验证的深度学习模型的肺结节良恶性分类模型准确性在79.5%-93.6%，而基于相同类型数据库的训练和验证的深度学习模型的分类准确性在68%-99.6%^[14]^，已经具有了较高的肺结节诊断的准确性。

在CT影像上，肺结节主要依赖于结节的大小和生长来区分良恶性，此外，CT影像还可以提供病灶的形状、空间复杂性和一系列其他“纹理”特征^[15]^。根据既往统计^[16]^，在临床实践过程中，AI辅助影像学诊断肺结节分类优势的主要体现在可以快速给出肺结节良恶性的判断，减少了放射科医生的工作量，提高了诊断的效率，并且AI算法能突出并呈现可疑成像特征区域给放射科医生，有利于减少肺结节分类的误诊率。

而AI技术算法相比传统人工肺结节分类的准确度尚需进一步提高。在LUNGx Challenge对肺结节分类的研究中^[17]^，AI组的曲线下面积(area under the curve, AUC)值低于放射科医师组(0.50-0.68 *vs* 0.70-0.85)，且3名放射科医生在统计学上的表现均优于最好的计算机方法，表明AI技术鉴别的肺结节准确度还无法替代人工，算法尚需进一步优化。

临床中发现在基于常规CT检查的诊断可能存在不少误诊病灶如大量的良性结节。这些病灶的确切病理性质在进行治疗或手术切除以前难以判断。而相较于CT，磁共振成像(magnetic resonance imaging, MRI)图像能够提供病灶的功能信息，如结节代谢速度，更适合某些病灶的定性与分类。因此，融合CT-MRI多种模态信息的肺癌诊断技术，尤其是通过AI的方法提取和融合不同影像针对肺癌病灶病理检测的有效影像特征，有望得到更加精准的肺癌诊断效果。同时，不同肺癌分期治疗后的生存率具有显著差异^[18]^。比如，经手术切除的Ia期中原位癌和微浸润腺癌其治疗后复发风险和5年生存率皆优于同期的浸润性腺癌^[19, 20]^。因此，通过探索融合多模态影像的智能诊断方法实现肺癌及其亚型的精准诊断对优化治疗方案设计和提高预后生存率都具有重要的临床意义。

## 5 肺结节病理分型对手术规划的指导意义

专家共识：现有数据支持：①部分低危(浸润前病变或贴壁样生长方式为主)的Ⅰ期非小细胞肺癌(non-small cell lung cancer, NSCLC)患者接受亚肺叶切除的预后不劣于肺叶切除者; ②含有高级别成分(包括实性生长模式、微乳头样生长模式以及复杂的腺样结构)的肺腺癌患者手术切除后预后不良(**共识强度：一致共识**)。由于术中冰冻和术后石蜡病理诊断肺腺癌亚型的一致性不够高，因此根据术中冰冻结果决定手术切除范围尚有争议。目前亟需新方法在手术前辅助诊断以指导后续治疗(**共识强度：一致共识**)。

JCOG系列研究发现部分低危(浸润前病变或贴壁样生长方式为主)的Ⅰ期NSCLC患者预后良好^[21]^，部分接受亚肺叶切除的Ia期NSCLC患者预后不劣于肺叶切除者。如JCOG0804研究^[22]^发现肿瘤最大径不超过2 cm且实性成分比例(consolidation tumor ratio, CTR)不超过0.25的肺结节接受亚肺叶切除者5年无复发生存率高达99.7%。JCOG0802证实对于直径≤2 cm、CTR > 0.5的周围型NSCLC肺段切除在总生存期(overall survival, OS)和肺功能方面显著优于肺叶切除。

然而，肺腺癌的某些亚型往往预后不良。国际肺癌研究联盟(International Association for the Study of Lung Cancer, IASLC)病理委员会关于肺腺癌的组织学分级中提出含有高级别成分(包括实性生长模式、微乳头样生长模式以及复杂的腺样结构)的肺腺癌患者预后不良。虽然气腔内播散(spread through air space, STAS)同样与不良预后相关，但是STAS与上述高级别成分高度相关，因此未单独列出^[23]^。有学者^[24]^提出根据术中冰冻结果指导手术切除范围，但是术中冰冻和术后石蜡病理诊断肺腺癌亚型的一致性不够高(κ=0.565)，术中冰冻诊断微乳头和实性成分的敏感性仅为37%和69%。

## 6 AI在肺结节病理分型预测中的作用

专家共识：AI依托深度学习与记忆可准确提取肺结节中有重要影响的微特征，具有无创、可捕捉肿瘤异质性和可重复性等优势，有望分级和预判GGN早期肺腺癌浸润亚型，为临床决策提供参考，但需要设计多中心、高质量数据集、前瞻性随机对照试验以进一步验证(**共识强度：一致共识**)。

随着胸部低剂量薄层CT筛查项目的广泛开展，越来越多的无症状肺部GGN被发现。GGN分为pGGN和混合GGN(mixed GGN, mGGN)。早期肺腺癌的影像学常表现为GGN，其预后与病理亚型显著相关^[25, 26]^。浸润前病变、微浸润腺癌和贴壁为主型腺癌的术后生存率较高，可考虑亚肺叶切除或随访观察^[27]^; 而如前所述，浸润性肺腺癌的某些病理亚型则提示肺叶切除的必要性。因此，临床迫切需要准确诊断GGN的病理亚型，决定随访管理、手术时机和手术方式的选择、辅助治疗、术后随访及预后评估等。肺穿刺活检因取材受限常常无法准确判断整体病灶的浸润性，且为有创检查，存在着一定的操作风险和结果的不确定性。传统CT影像判读多依据结节的特征判断病灶的侵袭性。然而，实际临床应用中，不同级别和资历的医师对上述影像特征的理解和认识存在着一定差异，判别能力也各不相同，且传统图像特征分析存在操作复杂、人为因素影响大、影像特征特异性不足等问题，影响临床图像判读的准确性。

为帮助医师提升影像诊断的准确性，减轻简单重复工作，减少漏诊及误诊对患者造成不良后果，AI应运而生^[28]^。借助计算机软件高通量地从临床影像中提取图像高维复杂特征，然后利用统计学或计算机学习的方法，对影像数据深度挖掘来定量分析肿瘤异质性，在高通量数据中发现肉眼无法分辨的影像学特征规律，已被逐渐应用于肺结节的行为预测，能较好鉴别以GGN为主要影像学表现的肺腺癌是否为浸润性病变，为早期诊断和个体化治疗提供临床依据^[29-35]^。

## 7 随访在肺结节诊疗中的价值

专家共识：根据肺结节基线检查特征拟定随诊方案，推荐采用高分辨率CT(high-resolution CT, HRCT)行小于1 mm的薄层扫描随诊，同时重建冠状位及矢状位以更完善地评估(**共识强度：一致共识**)。随诊方式建议按肺结节分类及风险分层进行区分。若结节具有生长性，可认为具有手术指征(**共识强度：基本一致共识**)。科学的随访方案可精确筛选病例，提高手术病例阳性率(**共识强度：一致共识**)。随访计划建议与患者进行共同决策(**共识强度：一致共识**)。

肺结节筛查阶段应使用薄层低剂量CT(low-dose CT, LDCT)进行，随后以HRCT进行进一步鉴别及随诊^[36]^。结合目前已有的国内关于肺结节筛查及随诊方法的文献总结^[37-40]^，再参照国外指南^[41-43]^，建议将肺结节分类分层管理。肺结节分为实性结节、部分实性结节和pGGN。按结节类型最多可分为高危、中危及低危结节。BTS指南建议使用Brock风险预测工具。如果恶性肿瘤的风险较低(< 10%)，建议进行影像学随访。但是，如果风险较高(> 10%)，建议考虑采用更具侵入性的诊断方法^[44]^。高危结节(直径 > 15 mm或直径8 mm-15 mm表现恶性特征的结节)建议积极进行多学科会诊进一步检查以鉴别诊断或积极进行手术治疗。中危结节(直径5 mm-15 mm无明显恶性特征的结节)若具有生长性或实性成分增加，则按高危结节处理^[45]^。若无改变可按3个月-12个月间隔随诊，建议至少随访5年，随后的随访计划应与患者共同决策。低危结节(直径 < 5 mm的结节)若具有生长性或实性成分增加则纳入高危结节，若无改变可按12个月-24个月间隔随诊，建议至少随访5年，随后的随访计划应与患者共同决策。若随诊过程中结节缩小或消失，可延长随诊间隔或终止随诊^[40, 46]^。多发结节者按最高危结节作为随诊方法基准^[40]^。美国国家癌症综合网(National Comprehensive Cancer Network, NCCN)指南根据结节大小对结节生长的定义不同。对于直径 < 15 mm的结节，生长定义为与基线扫描相比，任何结节或部分实性结节的实性部分的平均直径增加2 mm或更多。对于直径≥15 mm的结节，生长被定义为与基线扫描相比平均直径增加15%^[47]^。若结节具有生长性，则可以认为是有手术干预指征。除了线性增长外，还有计算体积增长和质量生长的方法，但这两种计算方法对其生长性的定义尚未能形成较好的标准^[48, 49]^。Veronesi、Nomori等^[49, 50]^研究表明，使用PET-CT来区分亚实性结节的良恶性是不合适的。PET对实性成分 < 8 mm的肺结节的敏感性较低^[47]^。

## 8 AI在肺结节多次随访数据的综合判断作用

专家共识：AI在肺结节多次随访数据中可协助评估肺结节体积、形态变化，对肺结节随访提供结节倍增时间变化、形态学改变等参考依据，进而制定个体化随访间期，但其具体适用范围有待进一步研究(**共识强度：基本一致共识**)。

针对同一肺结节不同时段的影像检查可提供结节倍增时间、结节形态学特征(如毛刺、分叶、密度变化)等信息为影像科医师判断结节性质提供参考。然而，除了肺结节性质差异导致的影像学特征动态改变以外，不同时期的影像检查中的同一结节亦可因检查体位、心脏搏动、吸气程度等的不同而导致结节大小的差异。近年来，AI在肺结节的性质判断和随访决策方面展露出较好的应用前景，但AI系统在肺结节随访领域的探索尚处于研究初期^[51]^。

据文献^[52]^报道，在利用电脑自动辅助系统对不同时间点肺转移瘤的图像识别中，同一结节的自动识别度可达82.4%，然而，当患者肺结节数目增加时，同一结节前后自动识别准确度随之下降。Ardila等^[53]^针对6, 716例NLST入组人群进行分析，结果发现当仅有单次CT影像数据时，AI系统较6位影像科医师判读可降低11%的假阳性率及5%的假阴性率; 而当有初次影像数据进行对比时，AI系统与影像科医师判读结果相仿，该研究结果提示AI系统可在肺癌筛查的多次影像检查中参与更多决策。在另一项研究^[54]^中，基于AI算法动态评估肺结节的两次影像检查亦可提高肺癌的诊断率。

除此之外，利用AI系统还可以指导肺结节的随访策略制定。近期一项研究^[55]^提示，当使用AI系统与Lung-RADS系统进行结合时，有约0.2%原Lung-RADS判定分级1级-2级的结节会升级至3级，而有30%的Lung-RADS 3级的结节会降至1级-2级，并可据此结果更改这部分人群的结节随访间隔，避免过度频繁的影像学检查或不必要的穿刺活检，显著减少了此部分人群的医疗费用。因此，应用AI辅助开展肺结节随访，可以协助制定更为科学、合理的随访策略，有望优化医疗资源分配。

## 9 AI在肺结节手术规划中的作用(三维重建)

专家共识：基于AI的三维重建技术对于提高手术的安全性和准确性具有重要的意义(**共识强度：一致共识**)。

AI依托神经网络深度学习可以进行肺结节良恶性判断及病理分型预测，有望实现术前肺癌及其亚型的精准诊断，从而优化治疗方案设计和肺结节手术规划。同时，借助AI三维可视化重建技术，可以提高手术成功率，实现精准切除，尤其对于亚肺叶切除手术的术前模拟规划。

目前，精准化的肺段切除术较为复杂，由于肺部血管、支气管较多，对医生解剖知识和操作技术的要求较高^[56]^。三维重建可以通过计算机图像处理技术对肺部各个组织器官进行自动重建^[57]^，其可视化结果可以全面地展示病灶的位置、大小、类别，同时也可分析病灶与肺动脉、肺静脉和支气管以及邻近组织的解剖和位置关系，因而可以为辅助医生进行术前规划提供一个有效的解决方案^[58, 59]^。进而，通过可视化与AI方法结合可以辅助医生精确定义肺段和楔形切除的范围和手术路径。因此，相关的研究对提高肺癌手术的安全性和准确性、保护患者术后肺部功能进而改善预后效果有重要的意义^[60]^。

## 附录

**Table:** 

**本共识编撰指导专家**	
**龙浩**	**中山大学肿瘤防治中心**
**陈克能**	**北京大学肿瘤医院**
**蔡开灿**	**南方医科大学南方医院**
**赵松**	**郑州大学第一附属医院**
**张春芳**	**中南大学湘雅医院**
**罗清泉**	**上海市胸科医院**
**李强**	**四川省肿瘤医院**
**李高峰**	**云南省肿瘤医院**
**胡坚**	**浙江大学附属第一医院**
**吴明**	**浙江大学附属第二医院**
**周清华**	**四川大学华西医院**

**执笔专家**	
**林勇斌**	**中山大学肿瘤防治中心**
**郑斌**	**福建医科大学附属协和医院**
**廖虎**	**四川大学华西医院**
**闫万璞**	**北京大学肿瘤医院**
**林志潮**	**江门市中心医院**
**李斌**	**兰州大学第二医院**
**汪路明**	**浙江大学附属第一医院**
**姜龙**	**上海交通大学附属胸科医院**
**赵泽锐**	**中山大学肿瘤防治中心**
**林耀彬**	**中山大学肿瘤防治中心**

**参与共识讨论的专家(按姓氏拼音排名)**	
**陈锋夏**	**海南省人民医院**
**陈捷**	**广东医学院附属医院**
**古卫权**	**佛山市第一人民医院**
**刘继先**	**北京大学深圳医院**
**马德华**	**浙江省台州医院**
**彭江洲**	**南方医科大学附属第三医院**
**田磊**	**联勤保障部队第九六〇医院泰安院区**
**汪潜云**	**常州市第一人民医院**
**王光锁**	**深圳市人民医院**
**王晓东**	**深圳宝安人民医院**
**肖海波**	**上海交通大学附属新华医院**
**许辰阳**	**赣州市人民医院**
**杨玉伦**	**郑州人民医院**
**叶新桥**	**赣州市立医院**
**于红**	**上海交通大学附属胸科医院**
**袁义**	**佛山市顺德区人民医院**
**张志锋**	**揭阳市人民医院**
**郑亮承**	**温州医科大学附属第一医院**
**周海榆**	**广东省人民医院**
**邹志强**	**联勤保障部队第九六〇医院**
